# Language development deficits and early interactive music intervention (BusyBaby): protocol description of a double-blind randomized controlled trial on the effectiveness of music on language development in infancy

**DOI:** 10.3389/fpsyg.2025.1699558

**Published:** 2026-01-05

**Authors:** P. Virtala, B. Aquilino, P. Nie, S. Navarrete-Arroyo, S. Stolt, K. Leutonen, M. Lauronen, T. Kujala

**Affiliations:** 1 Cognitive Brain Research Unit, Department of Psychology, Faculty of Medicine, Finnish Centre of Excellence in Music, Mind, Body, and Brain, University of Helsinki, Helsinki, Finland; 2 Department of Logopedics, Faculty of Medicine, University of Helsinki, Helsinki, Finland

**Keywords:** randomized controlled trial, dyslexia, language development, music intervention, infancy, neural speech processing

## Abstract

**Background:**

Infancy and early childhood lay the foundation for language and reading abilities. They are crucial for success in social relationships, education, and work life, but can be compromised by heritable conditions such as developmental dyslexia. Music activities have shown associations with auditory, language, and literacy skills, but randomized controlled trials (RCTs) have been lacking, especially in young and at-risk populations. This two-arm RCT evaluates the efficacy of an early music intervention for language development. As highly novel aspects, we investigate whether and how the efficacy is influenced by familial dyslexia risk and its genetic markers, as well as intervention timing (children’s age and developmental status), and mediated by expected social–emotional benefits of the interventions on the parent, child, and their interaction.

**Methods:**

A maximum of 200 infants will be recruited for the trial, with approximately 50% of them at familial dyslexia risk, and randomized to the experimental (music intervention) or control arm (circus intervention), where they will start a weekly 6-month intervention at the age of approximately 8–12 months. Outcome measures will be evaluated at baseline, at 6 months (after the intervention), and at a 1-year follow-up (1.5 years from the baseline). As primary outcome measures of language development, language abilities will be evaluated with standardized parental questionnaires (validated parental report instruments) and neural speech processing with auditory event-related potentials. Further outcome measures include, for example, standardized tests of language development, standardized parental questionnaires on social–emotional factors, neural processing of music, questionnaires and standardized tests of motor development, and dyslexia genetics (DNA sampling).

**Discussion:**

The present trial is expected to fill significant gaps in previous research on the effects of early-age musical activities on language development. By comparing a musical intervention to an equally active, social, structured, and pleasurable circus intervention in a randomized, controlled setting with pre- and post-assessments, the unique benefits of musical activities can be probed reliably. The trial can provide completely novel information on the moderators and mediators of the intervention outcomes, including participant characteristics such as dyslexia risk, age, and developmental stage, as well as social–emotional benefits of pleasurable group activities.

**Clinical trial registration:**

ClinicalTrials.gov; identifier NCT06261307.

## 1 Introduction

### 1.1 Background

Infancy and early childhood lay the foundation for the fundamental human abilities of language and literacy (Kuhl, 2004). When language and literacy development are deficient or delayed, the risks of negative outcomes in later stages of life increase significantly, compromising school and career paths and possibly causing mental health issues. This raises the need for early interventions. A prevalent cause of atypical language and literacy development is developmental dyslexia (Eden et al., 2016; Peterson and Pennington, 2015), a specific learning disorder related to reading. Dyslexia is heritable, with several identified candidate genes affecting early brain development (Kere, 2014). Thus, infants born to dyslexic parents are an especially vulnerable group for deficient or delayed language and literacy development. While early support for language development is valuable for all children, these at-risk children are particularly in need of supportive and preventive interventions.

Dyslexia is understood to stem from a phonological processing deficit, associated with structural and functional abnormalities in the left auditory cortex, which is especially important for speech processing (Eden et al., 2016; Giraud and Ramus, 2013; Vellutino et al., 2004). Brain research, including electroencephalogram (EEG) studies utilizing the event-related potential (ERP) technique, has shown that neural auditory and speech processing problems in dyslexia (Hämäläinen et al., 2013) can be detected in infants at familial risk (Thiede et al., 2019; Virtala et al., 2022) and can also predict future problems in language and reading-related skills (Cantiani et al., 2016; Kailaheimo-Lönnqvist et al., 2020; Leppänen et al., 2010; Navarrete-Arroyo et al., 2024a; Navarrete-Arroyo et al., 2024b; see Volkmer and Schulte-Körne, 2018 for review). Thus, neural auditory and speech processing in infancy can be seen as a promising predictive marker for language and literacy development. Infancy is also an important sensitive period for the development of speech processing: it is a time of immense neural plasticity that helps the infant acquire the phoneme representations of the native language (Kuhl, 2004). Therefore, supporting auditory and speech processing early on could be a promising route to diminish the risk of later difficulties in language and literacy.

It is widely acknowledged that music-based interventions can have benefits for health and wellbeing across the lifespan [e.g., report by the World Health Organization (Fancourt and Finn, 2019)]. Specifically, music practice is positively associated with enhanced speech processing, language development, and literacy skills (Kraus and Chandrasekaran, 2010; Tallal and Gaab, 2006). These benefits are proposed to stem from the overlap between music and language, particularly their reliance on auditory (temporal) processing [for reviews and proposed frameworks, see (Fiveash et al., 2021; Ladányi et al., 2020; Patel, 2012; Peretz et al., 2015; Tierney and Kraus, 2014); meta-analysis (Neves et al., 2022)].

Furthermore, in infancy, many parent–infant interactions are naturally musical, and musical activities are recognized globally as part of early parenting (Trehub, 2019). This makes music particularly promising for infant interventions. In young, even preverbal children, correlational and intervention research shows that playful and informal musical activities, such as shared musical activities at home and musical playschool (early childhood music education), are associated with improved auditory and language skills (Linnavalli et al., 2018; Putkinen et al., 2019; Papadimitriou et al., 2021). Looking at intervention studies in typically developing infants, particularly active and social music interventions, compared to social activity with passive or no music exposure, have been shown to improve early language development (Gerry et al., 2012) as well as neural processing of speech and music (Zhao and Kuhl, 2016; see also Benasich et al., 2014; Musacchia et al., 2017; Zhao et al., 2022). However, these infant intervention studies often suffer from methodological weaknesses, making it uncertain if the obtained effects are attributable to the music activities (Virtala and Partanen, 2018, for review). To date, very few studies have incorporated both pre- and post-intervention assessments to demonstrate intervention-related change in outcome measures; furthermore, these studies rarely utilize several complementary assessment methods [such as neural and behavioral measures (Gerry et al., 2012; Trainor et al., 2012)]. Furthermore, aiming for large sample sizes is particularly important in infant intervention studies, given the high variability typically observed in infant behavioral and neurophysiological measures (Robb et al., 2018). So far, previous studies reviewed here report sample sizes of only around 20 per intervention group. Most importantly, the authors are aware of no previous music intervention studies on infants’ language development where music-making was compared to another equally structured group activity. This is required in order to probe the unique effects of music on early language development.

In terms of intervention timing, the previous active music interventions for infants have taken place around 6–12 months of age (Gerry et al., 2012; Zhao and Kuhl, 2016; Zhao et al., 2022; Trainor et al., 2012). This choice is justified, as infants younger than this have limited abilities for active participation, and the most sensitive period for phonetic learning is understood to end around 12 months (Kuhl, 2004). However, it is possible that a slightly later timing of a music intervention may result in additional or alternative benefits for language development. For example, infants around 12 to 18 months who have already uttered their first words and progressed in fine and gross motor development may be more ready to actively take part in music-making, such as singing, clapping, or instrument playing, than younger infants. Finding the optimal age for an early music intervention for language development would require the inclusion of infants at various ages in the same study.

Previous intervention studies comparing music-making to other structured group activities in older, school-aged children have shown benefits of music on reading and related skills (Chobert et al., 2014; Moreno et al., 2009), specifically in children with dyslexia (Flaugnacco et al., 2015; see also Habib et al., 2016; Moreno et al., 2015; Overy, 2000; Overy, 2003). However, research-based evidence is scarce on whether musical interventions for infants and young children at familial risk of dyslexia or language disorders could support auditory and speech processing, phonetic learning, and early language development, thus ameliorating later language and reading deficits. To this end, our recent pioneering intervention study in 0- to 6-month-old infants at familial risk for dyslexia showed benefits of vocal music listening on neural speech sound processing, including phoneme discrimination, compared to instrumental or no music listening (Virtala et al., 2023). The enhancement of speech–sound discrimination in the vocal music listening intervention group was observed as enhanced positive mismatch responses (MMRs), infant ERPs reflecting auditory discrimination abilities. However, group differences were no longer evident at 28 months of age (Virtala et al., 2023). This may be attributable to the passive nature of the music exposure, as musical interventions that include active participation and social interaction seem to result in greater benefits, as reviewed above. Investigating their potential benefits in these at-risk groups calls for more research, as inherited risk factors may significantly moderate intervention outcomes. This could help in effectively targeting interventions to those children who can truly benefit from them.

In addition to enhanced auditory and language abilities, active and social music-making improves social–emotional development in infants and children (Gerry et al., 2012; Kirschner and Tomasello, 2010) and can support parent–infant interaction and parental wellbeing (Kostilainen et al., 2021). These broad social–emotional benefits of musical interventions are closely tied to the rewarding nature of music (Salimpoor et al., 2015) and might act as important mediators between musical activities and enhanced language development. However, these benefits are not unique to musical interventions and may also be achieved by other pleasurable group activities promoting social interaction. These benefits should thus be taken into account as potential mediators when evaluating the efficacy of both music and active control interventions on language development. Furthermore, ideally, the music and active control interventions should be at least somewhat balanced in their level and quality of social interaction. For example, in studies of older children, music interventions have often been compared with painting or visual arts (Flaugnacco et al., 2015). If the painting lessons contain less social interaction than the music lessons, this difference itself can contribute to the obtained benefits of music lessons for language and literacy.

To this end, based on the evidence presented above, we argue that active, social music-making in infancy and early childhood will result in improved language development through factors specific to music activities and by factors more generally important for early learning. The factors specific to music activities include particularly exposure to and (synchronous) production of musical and speech sounds, such as rhythms, melodies, rhymes, and singing. These result in (1) improved auditory/temporal processing as postulated, e.g., in Fiveash et al. (2021), but also (2) improved phonetic learning [as shown in Virtala et al. (2023)] that will benefit language development due to shared neural mechanisms. The more general factors important for early learning include the social setting and the pleasurable sensory–motor activity and stimulation, these two being also heavily intertwined and supported by the presence of the primary caretaker. Thus, we state that music intervention studies should introduce an active control intervention that shares these general factors enabling early learning while lacking the factors specific to music activities. This way, the studies can reliably probe whether music activities can have specific benefits on early language development over the benefits achieved by any structured social, pleasurable, and age-appropriate activity.

### 1.2 Objectives and research questions

The present study aims to fill important research gaps by investigating the effectiveness of an active, social music intervention compared to an equally active and social non-musical intervention (circus) for early language development. The participants will be infants ranging from eight to 12 months at the start of the intervention, with or without familial dyslexia risk. This will allow us to investigate how the infants’ age and developmental stage, as well as their familial risk factors, affect potential intervention outcomes. The comparators (intervention arms) were chosen and designed so that both are structured, social, and pleasurable, targeting important aspects of early development. Both activities are established and popular parent–infant hobbies in Finland and are therefore expected to be well-suited for the age group. The choice of family circus as an active comparator is supported by a recent scoping review concluding that circus interventions consisting of activities such as acrobatics, balancing, and juggling can benefit physical/motor and social–emotional development (Coulston et al., 2023). Thus, while the music and circus interventions share the general aims of promoting early learning and social interaction, they differ in their arm-specific aims (promoting musical vs. motor abilities, respectively). Language development is assessed with complementary behavioral and neural measures, namely standardized parental questionnaires (validated parental report instruments), tests on language abilities, and ERPs and MMRs to speech sounds and their changes.

As highly novel aspects, this trial aims to investigate how the intervention effects are modified by the participants’ age and developmental stage, as well as their inherited risk factors (i.e., familial dyslexia risk). It is further evaluated whether and how intervention arms differ in their social–emotional benefits and how the intervention benefits on language are mediated by the social–emotional benefits. These objectives are investigated in the following five main research questions (with relevant hypotheses referred to in parentheses):

*R1:* What are the effects of a social and active music intervention vs. a similar non-musical (control) intervention in infancy on language development (H1, H2)?

*R2:* How are the intervention effects in R1 influenced by the timing of the intervention (child’s age and developmental status, especially related to language and motor skills; H3)?

*R3:* How are the intervention effects in R1 modified by familial dyslexia risk (H4)?

*R4:* Are intervention effects in R1 mediated by the social–emotional benefits of the interventions, including infants’ social–emotional development, parental wellbeing, and parent–infant interaction (H5)?

*R5:* Do the two intervention arms have specific benefits on the abilities that are targeted directly (music processing in the music intervention, H6; motor abilities in the circus intervention, H7)?^1^

### 1.3 Trial design and hypotheses

**Figure 1:**
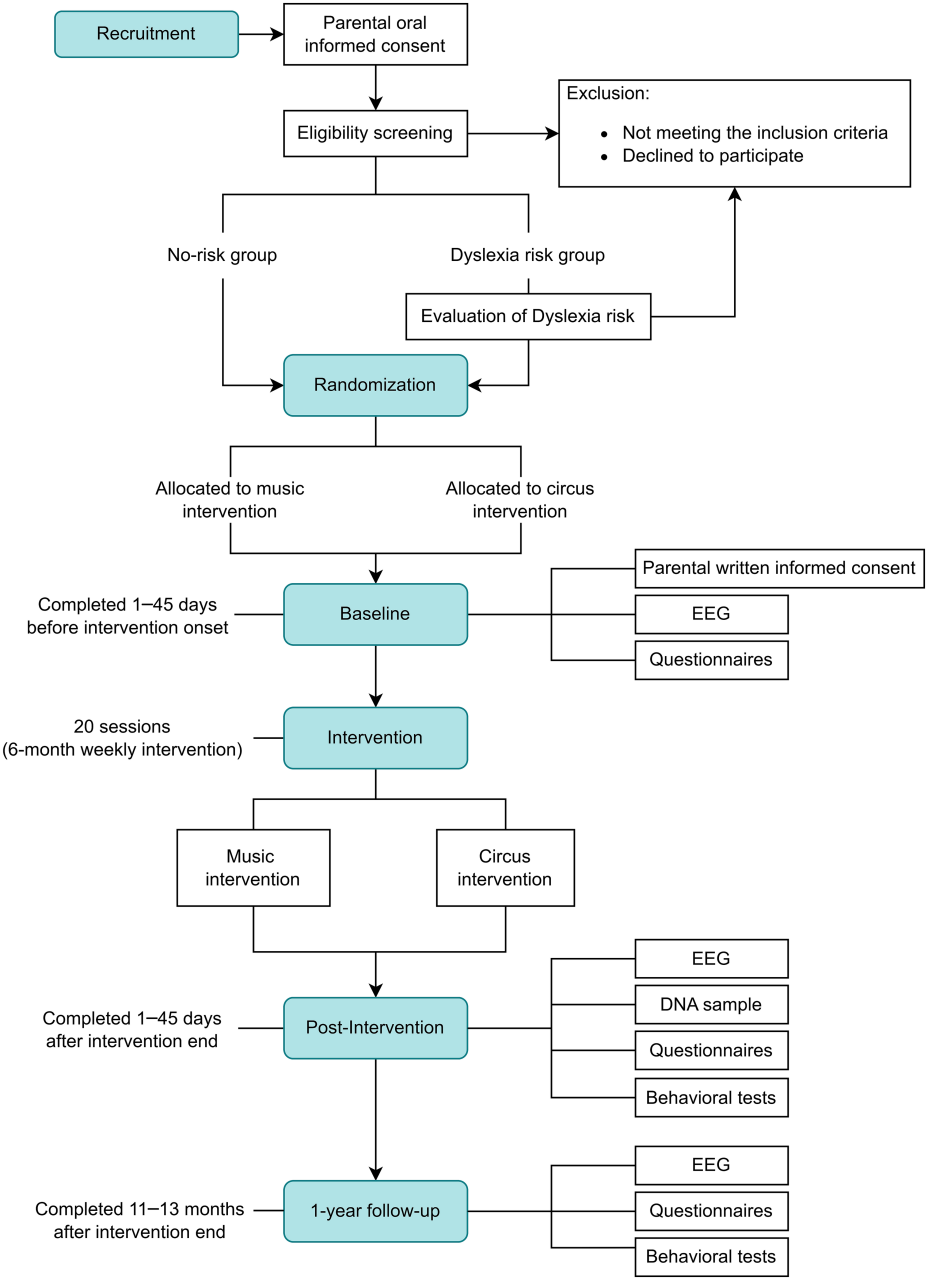
Flowchart of the randomized controlled trial.

This randomized controlled trial is conducted as a double-blind, two-arm parallel assignment design with the primary purpose of treatment, with primary and secondary outcomes assessed at baseline, post-intervention, and at follow-up approximately 1.5 years from baseline. The experimental arm will receive family music training (music intervention), and the control arm will receive family circus training (circus intervention). The study framework is a superiority trial with greater expected benefits from the experimental than the control arm. All participants are randomized to one of two interventions with a 50:50 allocation ratio, both consisting of approximately 6 months of weekly group sessions together with a parent (Figure 1: study flowchart).

The following main hypotheses are investigated:

#### 1.3.1 Primary outcomes

*H1:* Music intervention benefits language development.

It is expected that the music intervention group will show larger improvements in language abilities and neural speech processing between the baseline and post-intervention time points compared to the control intervention group. Language development will be evaluated with standardized parental questionnaires (validated parental report instruments), where a greater increase in the questionnaire scores is expected between the baseline and post-intervention assessments in the experimental (music) compared to the control arm (circus). Speech processing will be evaluated with ERPs and MMRs to speech sounds and their changes. ERPs and MMRs are hypothesized to display a more mature pattern in the experimental (music) than the control arm (circus) after but not prior to the intervention.

#### 1.3.2 Secondary outcomes

*H2:* Benefits of the music intervention are long-lasting.

It is expected that the experimental arm will show superior language abilities and more mature/enhanced neural speech processing compared to the control arm still at the 1-year follow-up assessment. In addition to the outcome measures listed in H1, the hypothesis will be probed with a standardized language test at post-intervention and follow-up assessments.

#### 1.3.3 Other/exploratory outcomes

*H3:* Intervention benefits depend on the age and developmental stage of the infants.

It is explored how infants’ age and important developmental milestones, such as walking without support and uttering first words at the start of the intervention, moderate the benefits on language development. For example, the benefits for neural speech sound processing may be larger the younger the infants start the music intervention (Kuhl, 2010); on the other hand, older infants might demonstrate more benefits for language development due to better readiness for active participation.

*H4:* Intervention efficacy may vary according to dyslexia risk.

It is explored whether the intervention benefits for language development are moderated by familial dyslexia risk. For example, the dyslexia-risk group can benefit more if ceiling effects are assumed in the no-risk group; on the other hand, genetic risk may compromise the risk group’s ability to benefit from the intervention (e.g., via reduced neuroplasticity in speech and language-related networks). These exploratory hypotheses are studied by confirming the parental dyslexia status and by investigating allele groupings in single-nucleotide polymorphisms of susceptibility genes associated with language, reading, and developmental dyslexia, analyzed from DNA samples of the infants and their biological parents.

*H5:* Social–emotional benefits of the interventions mediate the benefits for language development.

It is explored whether the experimental arm demonstrates greater social–emotional benefits than the control arm and whether these benefits mediate the benefits for language development. Social–emotional benefits are evaluated at the three assessment points with standardized parental questionnaires on infant and early childhood temperament, social–emotional/behavioral problems and delays, as well as parental wellbeing, stress, and interaction with the child.

*H6:* The music intervention will benefit music processing.

It is hypothesized that the development of music processing will be superior in the experimental (music) group compared to the control arm (circus), due to the main teaching aims of the music intervention. To this end, neural music processing will be evaluated with ERPs, particularly MMRs to changes in a melody, recorded at the three assessment points, and is expected to be more mature in the music group compared to the circus group after but not before the intervention. At follow-up assessment, behavioral musical abilities are additionally assessed with a custom-designed behavioral test battery, and better performance is expected in the music group compared to the circus group.

*H7:* The circus intervention will benefit motor abilities.

It is hypothesized that motor development, particularly gross motor development, is greater in the control (circus) arm than in the experimental arm (music) between the three assessment points, due to the main teaching aims of the circus intervention. Motor development is evaluated with customized parental questionnaires at baseline, post-intervention, and follow-up assessments, as well as with a standardized test at the post-intervention and follow-up assessments.

## 2 Methods and analysis

### 2.1 Participants

#### 2.1.1 Participant recruitment and consent

The participants are infants from Finnish families from the greater Helsinki region, recruited between March 2024 and September 2025 by distributing paper flyers and digital advertisements via the project website, social media (Facebook, Instagram), local maternity clinics, and public events and lectures. Families can express their interest in participation via an electronic form on the project website or by contacting the trial coordinator via the project email. Participation is expected to be appealing to families due to the intervention, which is offered at no cost to all participating families and is similar to a self-paid infant–parent hobby. In order to achieve a sufficient sample size (see 2.1.3), recruitment will continue for approximately 1.5 years.

The trial coordinator screens the families for eligibility and the need for parental dyslexia testing (see 2.1.2) on the phone with the parent and, after receiving oral consent for participation from the parent, conducts stratification, randomization, and allocation of eligible infants to the two intervention arms (see 2.2).

One or both parents of the infant will be provided with a participant information sheet and data protection notice and will sign written informed consent for participation before starting the baseline assessment. This study is conducted at the University of Helsinki, Finland. The study has been approved by The University of Helsinki Research Ethics Committee in the Humanities and Social and Behavioral Sciences with the title “Infant and toddler age hobbies as support for language development (BusyBaby)” (public title translated from Finnish), and preregistered at ClinicalTrials.gov with the title “Language Development Deficits and Early Interactive Music Intervention—a Randomized Controlled Trial (BusyBaby)” (scientific title; NCT06261307, February 7th, 2024). Any important protocol modifications will be reported at the preregistration site.

#### 2.1.2 Eligibility criteria

General inclusion criteria for the infants are that they must be 8–12 months old at the start of the intervention (recruited between 0 and 11 months) and must be born healthy and at term (gestational age of at least 37 weeks and birth weight of at least 2,500 g). Additionally, at least one caregiver living with the infant is required to be a native speaker of Finnish and to speak Finnish to the infant. General exclusion criteria for the infants include medication affecting the central nervous system, sensory deficits, and serious health conditions.

Families with (risk group) and without developmental dyslexia in one or both biological parents (no-risk group) are recruited. In the risk group, parental dyslexia is confirmed at enrollment in the study. To be included in the risk group, one or both biological parents must (1) suspect or state that they have dyslexia and (2a) either have a recent (< 5 years) clinical statement of dyslexia or, if it is not available, (2b) demonstrate reading problems since childhood in a clinical interview and current reading problems based on below-norm performance in a test battery [Finnish standardized test measuring speed and accuracy of oral text, word, and pseudo-word reading, and writing speed (Nevala et al., 2006)]. Parents who report clear reading problems in childhood and dyslexia in biological relatives are accepted into the risk group as compensated dyslexics, even with normative performance in the test. Exclusion criteria in the risk group include a diagnosis of a neurodevelopmental disorder other than dyslexia or developmental language disorder in either of the biological parents and, in the dyslexic parent, brain trauma in childhood that may indicate a non-heritable cause for the reading deficit, or an individualized school curriculum that may indicate broader developmental deficits.

In the no-risk group, exclusion criteria include suspected or diagnosed dyslexia or developmental language disorder in either of the biological parents, and a diagnosis of a neurodevelopmental disorder or severe neurological or psychiatric disorder in either of the biological parents. In both groups, at least one biological parent must be available to provide background information for the study. If only one biological parent is available, eligibility screening related to inherited risk factors is conducted based only on that parent.

#### 2.1.3 Sample size

The target maximum sample size is *N* = 200 infants. To reach a balanced sample, the aim is to recruit 30–50 participants for each stratum (see 2.2). A power analysis indicated that 50 participants per intervention arm are needed to evaluate the primary outcome measures, given a repeated measures ANOVA with a two-within (time of assessment: baseline vs. post-intervention) x two-between (intervention arms) design, a small effect size, Hedge’s g of 0.31 [based on the meta-analysis (Neves et al., 2022)], an alpha level of 0.05, a power of 0.8, and an expected correlation of 0.7 between the repeated measures.

Based on our previous longitudinal intervention study [see Virtala et al. (2022), Virtala and Partanen (2018), and Virtala et al. (2023), where 76% of the original sample recruited at birth participated in EEG and behavioral tests at 28 months], and given the engaging and rewarding nature of both intervention arms and the lack of a passive control group, we expect a low drop-out rate of a maximum of 20% for the primary outcome measures, including baseline and post-intervention assessments. This percentage includes families who will refuse participation before completing post-intervention assessments or who will not successfully complete some of the baseline or post-intervention assessments. Therefore, we assume that a sample size of *N* = 120 will be sufficient for reliably evaluating the primary outcomes of the interventions in the repeated measures ANOVA framework. However, it is relevant to note that this sample size is not optimal for probing the interaction of intervention arm x time in the linear mixed models or for the additional analyses (including, e.g., subgrouping based on participant characteristics or mediation analyses, see 2.9). To additionally calculate sufficient sample sizes per intervention arm for the planned linear mixed models, specifically considering the studied interaction of intervention arm x time, simulations were run according to Caldwell et al. (2022) and Kumle et al. (2021). These simulations suggested a larger required sample size of 100 per arm in order to ensure a power of 0.8, considering the values described above. We therefore aim for a sample size between 120 and 200, as large as practically possible within the 1.5-year time frame for participant recruitment.

### 2.2 Randomization and allocation

Randomization in the study is conducted as *stratified block randomization* with four strata and a block size of four. The strata are defined according to dyslexia risk status and infant gender as follows: dyslexia-risk boys, dyslexia-risk girls, no-risk boys, and no-risk girls [as dyslexia risk and infant gender are expected to have a large effect on the primary outcome measures, e.g., Snowling and Melby-Lervåg, 2016].

For each stratum, block randomization is completed by randomly permuting a list of four-letter combinations consisting of AABB, where A = experimental arm (music intervention) and B = control arm (circus intervention), in order to reach balanced group sizes in the two arms. This also ensures that new music and circus intervention groups can be started at the same time, as new participants will be randomized to the two arms at a similar pace during recruitment. Author PV produced the randomization sequence by creating a list of 200 random permutations of AABB with MATLAB v. R2023a at the onset of participant enrollment, and chronologically labeled them according to block (1, 2, 3…50) and stratum (a, b, c, d); however, this randomization sequence is not concealed from the trial coordinator who assigns the participants to the intervention arms. The trial coordinator is advised to enroll new participants and assign them to the intervention arms in the order that they are successfully screened for eligibility and express oral consent. In situations where there are more such families than available spots in the intervention groups or the strata have large differences in sample sizes (> 10 infants), the trial coordinator is advised to first enroll participants in the strata with smaller sample sizes and to start enrollment from participants who help to balance the mean age of that stratum close to 10 months of age.

Blindness of the researchers to the randomization has been ensured by assigning a dedicated member of the research team as trial coordinator. The trial coordinator screens participants for eligibility and assigns them to the two intervention arms according to the randomization sequence. When the evaluation of dyslexia risk has to be conducted, the trial coordinator will assign the evaluation to one of the outcome assessors, and that assessor will not participate in future outcome assessments for that family (as their blindness to the participant’s dyslexia risk status has been violated). The trial coordinator will not act as an outcome assessor or intervention instructor and will keep track of any blindness violations.

Blindness of the participants to the trial design is ensured by presenting the study as evaluating the effect of early-age hobbies on language development, without either intervention being a control arm. To evaluate how successful this was, participating families are queried about their expectations regarding the benefits of the two intervention arms on the primary outcome measures (see Supplementary material).

### 2.3 Interventions

Both intervention arms consist of 20 weekly sessions of 45 min, organized at fixed times and locations in fixed groups of 5–10 parent-infant dyads and an experienced instructor. The intervention arms share the general goals of supporting the infants’ holistic development and parent–infant interaction. Additionally, they have arm-specific teaching goals as specified below. The content, structure, and goals of the interventions, as well as intervention instructors, venues, and materials, are described in more detail in the Supplementary material.

#### 2.3.1 The experimental arm (music intervention)

The music intervention is planned based on the practices of Finnish early childhood music education and consists of social, structured, and playful group sessions that involve joint singing, playing with musical instruments, and moving to and listening to music. Based on common age-appropriate practices of Finnish early childhood music education, rhythmic exercises such as tapping, clapping, and playing percussion instruments (claves, maracas, etc.) with infants and parents together, as well as joint singing (mostly by parents), are emphasized in the sessions. Their use is supported by previous research, for example, on the benefits of rhythmic exercises on literacy in dyslexic children (Flaugnacco et al., 2015) and on the benefits of vocal music exposure on speech processing (Virtala et al., 2023). The arm-specific aim is to support the development of musical abilities.

#### 2.3.2 The control arm (circus intervention)

The circus intervention consists of social, structured, and playful group sessions that involve acrobatics and other age-appropriate motor exercises with the caregiver, familiarizing participants with the art and equipment of circus and acrobatics. The use of music is limited to occasional background music to set the mood and support the lesson theme (see Supplementary material), so that movement is not synchronized with it. The arm-specific aim is to support the development of motor abilities as well as bodily control and awareness.

### 2.4 Adherence and commitment to the study and concomitant activities

Participating families are rewarded for their participation in all phases of the study (baseline, post-intervention, and follow-up assessments) with a small book gift and snacks for the infant, and they are compensated for reasonable travel costs to the research laboratory upon request, in order to promote participant retention and complete follow-up. The parents can also receive oral feedback on their infant’s language and motor performance after the post-intervention and follow-up assessments.

Commitment to participate in the intervention sessions is facilitated by offering families a choice regarding the time and location of their intervention group whenever possible (i.e., when several groups in the right intervention arm have available spots at the time when the infant is in the desired age range). This freedom of choice will not affect their arm allocation but can influence which families participate in the morning versus evening groups. As the time of day may potentially influence intervention efficacy, it will be considered in the analyses of the primary outcomes.

During the interventions, the instructors continuously keep track of non-attendances and inform the trial coordinator in case of two or more consecutive no-shows. In this case, the trial coordinator tries to reach the family and encourage them to attend the sessions as often as possible. Discontinuation of the intervention occurs at the participant’s request.

In order to keep track of the reasons for low adherence, parents are asked to inform the intervention instructor of their absence from the sessions and the reason for it. The most common reasons for non-attendance are also queried from the families at the end of their intervention. The aim is to assess primary outcomes from all participants irrespective of intervention adherence (intention-to-treat analysis) and further investigate the effect of adherence on the primary outcomes (see 2.9). For per-protocol analyses (see 2.9), criteria for successful participation are defined based on the number of attended sessions out of a total of 20 and the total attended duration of the intervention (quantified as the period between the first and the last attended session).

During the intervention, the participating families are requested to refrain from concomitant similar activities. This means regular participation in family musical playschool, family circus, as well as acrobatics, dance, and sports classes for infants and toddlers. Participation in such classes is allowed before and after the 6-month intervention, and participation in other concomitant leisure activities (for example, baby swimming classes or classes targeted to the parent where the infants can be present, such as postpartum yoga) is always allowed. All other concomitant care, including interventions (for example, speech therapy), is permitted throughout the entire study and is inquired about from the families in questionnaires (see 2.5.4).

### 2.5 Outcomes

Outcomes of the trial are evaluated with three assessment points: at baseline (before intervention), post-intervention (six months from baseline, end of intervention), and follow-up (1.5 years from baseline). Data collection carried out by the outcome assessors takes place at the University of Helsinki research laboratories. Outcome assessors are trained graduate students in psychology and speech and language pathology, supervised by a licensed clinical psychologist (PV, in collaboration with a licensed speech and language pathologist, SS). All questionnaire data are collected online via REDCap (Research Electronic Data Capture).

#### 2.5.1 Primary outcomes

As primary outcomes, the children’s language development will be evaluated at baseline and post-intervention. This includes evaluating language abilities with two standardized parental questionnaires (validated parental report instruments) and speech processing with auditory ERPs and MMRs. The parental questionnaires and the neural measures (ERPs and MMRs) are expected to provide complementary information on early speech and language development.

##### 2.5.1.1 Language abilities

Language abilities will be assessed using two validated parental report instruments: The Infant–Toddler Checklist of the Communication and Symbolic Behavior Scales Developmental Profile [CSBS ITC (Wetherby and Prizant, 2002); Finnish version (Laakso et al., 2011)] and the standardized Finnish long form version of the MacArthur Communicative Development Inventories [Words and Gestures form, MCDI (Fenson et al., 1993); Finnish version (Lyytinen, 1999), Finnish Long Form version of the Communicative Development Inventories, FinCDI-LF]. Both methods have been normed for Finnish children and have shown to provide reliable information on the language skills of children [CSBS ITC (Laakso et al., 2011); FinCDI-LF (Lyytinen, 1999; Stolt et al., 2009)]. The CSBS ITC is a brief screening tool with three subscales (social, speech, and symbolic subscales) and a total score. The Words and Gestures form of the FinCDI-LF provides comprehensive information on children’s early receptive and expressive lexicons as well as on the development of early gestures. It gives scores separately for expressive and receptive vocabulary size and for the gestures scale for non-verbal communication.

##### 2.5.1.2 Speech processing

In a multi-feature oddball paradigm with three deviant types embedded in a speech sound stream [speech sound paradigm described, e.g., in Virtala et al. (2022)], auditory ERPs are recorded with EEG in a passive listening condition at baseline and post-intervention assessments. Auditory ERPs in response to repeating standard stimuli (obligatory responses P1, N2) and occasional deviant stimuli (MMRs) are extracted from the EEG. Peak latencies, mean amplitudes, and hemispheric lateralization of these responses are quantified from a fronto-central region of interest.

#### 2.5.2 Secondary outcomes

##### 2.5.2.1 Language abilities

The standardized parental questionnaires (validated parental report instruments) CSBS ITC and MCDI/FinCDI-LF 16–30 mo will be re-administered to evaluate the language abilities at the follow-up assessment (Wetherby and Prizant, 2002; Laakso et al., 2011; Fenson et al., 1993; Lyytinen, 1999). The MCDI/FinCDI-LF 16–30 mo provides the size of expressive vocabulary and the mean length of the three longest utterances (in morphemes). The standardized language assessment test battery *Reynell Developmental Language Scales III (RDLS-III)* (Edwards et al., 1997; Reynell and Huntley, 2001); presented to the participants in Finnish (Kortesmaa et al., 2001) is conducted at post-intervention and follow-up assessment points and provides scores for expressive and receptive language abilities. It is relevant to acknowledge that at the post-intervention assessment point, the participants are still below the age range of the RDLS-III (younger than 2 years), and therefore, the scores obtained in this assessment will be treated with caution.

##### 2.5.2.2 Speech processing

The speech sound paradigm described in 2.5.1.2 is re-administered with an identical protocol at the follow-up assessment.

#### 2.5.3 Exploratory/other

##### 2.5.3.1 Social–emotional factors

Three standardized parental questionnaires will be administered at all three assessment points to evaluate infant and early childhood temperament [Finnish translations of the Infant Behavior Questionnaire Revised short form, IBQ-R-sf (Putnam et al., 2014); Early Childhood Behavior Questionnaire short form, ECBQ-sf (Putnam et al., 2006, 2010)], social–emotional/behavioral problems, and delays in social–emotional competence [Finnish translation of the Brief Infant–Toddler Social Emotional Assessment, BITSEA (Briggs-Gowan et al., 2004)], as well as parental wellbeing, stress, and interaction with the child [Finnish translation of the Parenting Stress Index Short Form, PSI-sf (Abidin, 1995)]. The IBQ-R-sf and ECBQ-sf give scores for Negative Affectivity, Surgency/Extraversion, and Orienting/Regulation/Effortful Control. The PSI-sf consists of three factors: parental distress, parent–child dysfunctional interaction, and difficult child, as well as a total score. BITSEA consists of Problem and Competence scores.

##### 2.5.3.2 Dyslexia risk and genetics

Parental dyslexia status is confirmed as part of the eligibility screening (see 2.1.2), coded in the database, and used as a stratification variable. DNA sampling is conducted from saliva (infants, parents) or blood samples (parents) at the post-intervention assessment (or at another assessment phase, according to the preferences of the family). Allele groupings in single-nucleotide polymorphisms (SNPs) of susceptibility genes associated with language, reading, and developmental dyslexia are analyzed from the sequenced DNA and used to form subgroups.

##### 2.5.3.3 Musical abilities

At follow-up assessment, musical abilities will be investigated with a custom-made musicality test battery (the Music, Mind, Body, and Brain Music Battery, MMBB-MB; details of the battery will be reported in a separate publication), including melody (scale) and rhythm discrimination abilities, sensory–motor synchronization skills (including both whole-body movement and finger tapping), singing abilities, and perceived emotions in music.

##### 2.5.3.4 Music processing

In a melodic multi-feature paradigm with several musical deviants embedded in a short melody [paradigm described, e.g., in Tervaniemi et al. (2022)], auditory ERPs are recorded with EEG in a passive listening condition at all three assessment points. The ERPs in response to repeating standard stimuli (obligatory responses P1, N2) and occasional deviant events (MMRs) are extracted from the EEG. Peak latencies, mean amplitudes, and hemispheric lateralization of these responses are quantified from a fronto-central region of interest.

##### 2.5.3.5 Motor abilities

Fine and gross motor development will be evaluated with a custom-made parental questionnaire at all three assessment points and with a standardized test [Motor scale of the Bayley Scales III (Bayley, 2006), in Finnish] that includes sub-scales for fine and gross motor abilities at post-intervention and follow-up assessment points.

#### 2.5.4 Background variables and parent-reported activities

At baseline, a custom-made parental questionnaire is administered to collect basic information on the following background variables: child’s age, gender, family structure, living situation and daycare situation, language background and exposure, genetic risk factors for neurodevelopmental, neurological, and psychiatric conditions, socioeconomic status (indicated by parental education and current employment status), physical growth (weight and height at birth and their development), as well as healthcare and illnesses of the child. At post-intervention and follow-up assessment points, possible changes regarding family structure, living and daycare situation, language background and exposure, genetic risk factors, as well as physical growth (height and weight) and healthcare and illnesses of the child will be inquired.

At all three assessment points, custom-made parental questionnaires are additionally used to collect information on the frequency and intensity of the child’s musical activities (including joint singing, dancing, moving to music, and playing instruments at home, music listening, and exposure to music at home), musical background of the parents (including preferences, training, and listening habits), home literacy environment (including the amount of reading by parents, the amount of reading aloud to the child, the amount of children’s and adults’ books at home, frequency of library visits, and audio book listening), screen time, and all regular hobbies that the child attends.

### 2.6 Trial timeline

Recruitment started in March 2024, and the first family was enrolled in the study after randomization on 13 June 2024. Recruitment ended on 30 September 2025. The interventions are conducted between September 2024 and March 2026 (estimate). Altogether, 10 music interventions and 10 circus interventions will be conducted so that one music intervention and one circus intervention start nearly every month between September 2024 and October 2025 (estimate). The planned duration of all interventions is 5–7 months; however, all interventions consist of 20 weekly sessions with a fixed time (the number of canceled sessions and break weeks varies between interventions for practical reasons). Outcome measures will be collected at baseline (between August 2024 and September 2025), 6 months (post-intervention; between March 2025 and March 2026, estimate), and 1.5 years (follow-up, one year after post-intervention; between March 2026 and April 2027, estimate). Thus, data collection for primary outcome measures is estimated to be completed by March 2026 and for all outcome measures in April 2027.

By the end of recruitment and baseline assessments on 30 September 2025, 184 participants had been randomized to the study after providing oral consent. Of these, 154 participants had attended the baseline assessments and provided written informed consent (separate n’s according to strata: dyslexia-risk boys/dyslexia-risk girls/no risk boys/no risk girls *N* = 32/42/41/39; according to intervention arm: music/circus *N* = 75/79) with an average age of 10.17 months (SD 1.43, range 6.73–13.13; according to intervention arm: music/circus 10.18/10.14 months) at the first intervention session. Participants who were unable to attend the intervention sessions after providing written consent were nevertheless invited to all assessment points (see 2.9) unless they refused participation altogether. By the end of recruitment, one participating family with written consent had refused participation after the baseline assessment.

### 2.7 Data management

The data involve digital and paper format data and biological samples. All digital data are stored on a University of Helsinki network drive in a group folder accessible only to the research group members and backed up by the university’s IT center. Digital data are stored in password-protected relational databases where they are transferred directly from the REDCap (questionnaires) or entered manually by the outcome assessors (behavioral test scores, EEG measurement protocols). All paper form data and biological samples are stored in locked premises and locked cabinets that can only be accessed by essential personnel of the research team. A collaborating laboratory (Folkhälsan Research Centre) will store the biological samples (saliva samples) and send them in pseudonymized form (without direct identifiers) to another laboratory for genetic laboratory analysis (DNA sequencing and targeted genotyping; procured as a contracted service); remaining samples will be returned to the collaborating laboratory for long-term storage (see below).

Data pseudonymization is performed during data collection, and all data analysis is conducted without direct identifiers. Direct identifiers are replaced by codes and stored separately in password-protected files. The passwords are only available to research team members who essentially need the information (e.g., contact information of the parents is only accessible to outcome assessors and the trial coordinator). Only the trial coordinator and, as a safety procedure, author PV, have direct access to the separate password-protected files that contain information on the arm allocation of the participants. This information will be included in the password-protected relational databases together with the rest of the digital data but in an indirect manner, so that it cannot be revealed accidentally. Personal information and all records of interested families not enrolled in the study will be destroyed by the trial coordinator by the end of participant recruitment.

Data with long-term value are stored in locked cabinets and secure servers of the University of Helsinki and (in the case of the biological samples) Folkhälsan Research Centre for 10 years from the onset of the project, and after that for 15 more years without direct identifiers. The principal investigator is responsible for data publication, data protection, and information security. Only the members of the research team have access to the final trial dataset, and all research team personnel are trained in methodology and laboratory protocols.

### 2.8 Risk assessment and monitoring

Participation in the interventions is expected to have no adverse effects on the health, wellbeing, or safety of the participating families. Therefore, a data monitoring committee has not been appointed. Nevertheless, intervention instructors are asked to report all negative occurrences in their intervention groups after each session, including, for example, minor accidents, crying or negative emotions, and conflicts between the participants (see Supplementary material). Additionally, all negative feedback or concerns raised by the parents that the intervention instructors, researchers, outcome assessors, or trial coordinator receive during the trial will be collected and stored by the trial coordinator.

Challenges in participant recruitment or attrition are expected to be minor, particularly regarding the primary outcomes (evaluated directly before and after the intervention) for three main reasons. Firstly, participation in the trial is expected to be engaging and rewarding, as pointed out in section 2.1.3. Secondly, several actions are taken to support adherence and commitment to the study, as described in section 2.4. Thirdly, the follow-up period of 1.5 years in total is considered relatively short. Challenges related to data loss are further minimized by using complementary outcome measures. For example, while EEG recordings may not always be successful in infancy and early childhood due to movement artifacts and participant refusal, parental questionnaires will still provide valuable information. Nevertheless, participant drop-outs and no-shows are carefully monitored (see also Supplementary material).

The following measures have been taken to ensure intervention fidelity and arm equivalence. The interventions have been planned in advance, and their execution is based on detailed lesson plans. Teachers are asked to report any deviations from the lesson plan or duration after each lesson. The teachers have planned the lessons together and meet regularly (1 h/month) during the interventions to harmonize their practices as much as possible. The authors PV and BA conduct lesson observations for all teachers. For more information, see Supplementary material.

### 2.9 Data analysis

All analyses will preferably be conducted with intention-to-treat analysis, including all participants regardless of attendance rate and whether they completed the intervention. However, when deemed necessary, for example, due to the amount of missing data, the intention-to-treat analyses can be complemented by per-protocol analyses, i.e., with only those participants included who successfully completed the intervention (see 2.4).

In the primary analyses, the efficacy of the trial will be evaluated by investigating changes in the primary outcome measures between baseline and post-intervention assessments, based on an interaction between time (baseline vs. post-intervention) and intervention arm (music vs. circus).^2^ Main analysis methods will be linear mixed models and repeated-measures ANOVAs, with statistical significance defined as *p* < 0.05 and appropriate adjustments correcting for multiple comparisons. Linear mixed models are well-suited for accommodating missing data that may occur between assessment points through the use of maximum likelihood estimation, which allows all available data to contribute to the estimation of model parameters under the assumption of missing at random.

Additional secondary and exploratory analyses will be conducted. The possible moderating effects of intervention timing on the primary outcomes will be investigated by adding participant age and developmental status at intervention start as covariates or fixed factors to the primary analyses.^3^ The risk of dyslexia will be included in the statistical models as a fixed factor to account for its moderating effects on the primary outcomes, and additional stratification will be made based on both the severity of parental dyslexia and genetic background (SNPs associated with dyslexia and language development will be entered as fixed factors, with children categorized into risk and non-risk subgroups according to their allelic variation). The possible mediating role of social–emotional factors on the primary outcomes will be analyzed. To further explore factors moderating intervention efficacy, the effects of intervention timing (morning or evening), adherence to the intervention (attendance rate), motivation towards the intervention (see Supplementary material), as well as the social structure and atmosphere of the intervention (see Supplementary material) will be additionally considered in the analyses of primary outcomes. Similarly, the role of home activities, especially those related to music and literacy, as well as hobbies that the child participates in, will be taken into account in the primary analyses. Further analyses will be conducted to investigate potential effects of the intervention on intervention-specific outcomes, including neural music processing and musical abilities (music intervention) and motor abilities (circus intervention).

For all statistical analysis models, we will examine the necessary assumptions using appropriate diagnostic tests. Furthermore, if non-linear patterns of change in the outcomes are indicated across the three assessment points, we will consider the use of, for example, structural equation modeling approaches to more flexibly capture individual differences in change trajectories.

## 3 Discussion

This protocol describes an ambitious attempt to probe the effects of early musical interventions on language development with a multi-methodological and large-sample RCT. By randomly allocating participants to two active, structured, social, and pleasurable interventions and studying changes in language abilities between baseline and post-intervention time points, we aim to tease apart the unique effects of music activities on early language development. We aim to conduct a double-blind trial in which participants in neither arm should consider their intervention a control or placebo group. We expect this to minimize confounding factors related to participants’ motivation and expectations on the primary outcomes.

In addition to serving as a methodologically rigorous replication of previous pioneering infant music intervention studies, we expect the trial to provide answers to several completely novel research questions. Most importantly, these concern (1) how familial dyslexia risk and its genetic underpinnings moderate the intervention outcomes, (2) how intervention timing in terms of infant age and developmental stage moderates the intervention outcomes, and (3) how broad social–emotional benefits act as mediators for the intervention outcomes. Results from the trial can contribute markedly to designing optimal and efficiently targeted interventions for early language development.

### 3.1 Limitations

The present trial is conducted without a no-intervention (passive) control arm. Thus, in the case that no group differences are observed in the primary outcome measures at post-intervention, we are not able to conclude if both interventions still showed benefits compared to no extra activity. The lack of a no-intervention (passive) control arm was still considered an important ethical choice, as preventing the no-intervention control arm families from participating in similar kinds of activities at their own cost would have been both ethically questionable and practically challenging.

Furthermore, although participating families were advised to avoid engaging in other similar regular activities between the baseline and post-intervention assessments, it is still possible that many families will attend music, circus, or other similar hobbies, confounding the effects of the studied intervention. Similarly, home activities such as shared music-making are potential confounders of intervention effects, particularly because their intensity and frequency can be much higher than in the intervention, and their content can be very similar to that of the interventions. These risks can be partly mitigated by asking the parents to report their participation in all parent–infant hobbies and similar at-home activities at all three assessment points and considering them in the analyses. If participation in one intervention arm increases the frequency of similar activities at home, it can also enhance the obtained intervention benefits.

It is important to note that the 4-month age range of the participants will introduce considerable variation in the baseline and post-intervention outcome measures, making it more challenging to obtain significant intervention effects, especially if the benefits are small. However, participant allocation to the intervention arms is done so that the mean age remains similar in the two arms. The age range was also agreed upon with the intervention instructors to ensure that it would not compromise the practical implementation of the interventions, i.e., that children at both ends of the age range would be able to participate in the lessons in a meaningful way with the help of their parents. While participant age intentionally varies in the present trial, intervention intensity and duration are fixed to one weekly lesson for approximately six months. Therefore, evaluating the optimal intensity and duration for this kind of intervention is beyond the scope of this trial.

Due to the young age of the participants at baseline, the assessment of language abilities is based solely on parental questionnaires (validated parental report instruments). This is not optimal for the reliability of the language assessment and may particularly affect the evaluations of dyslexia-risk infants, as their dyslexic parents may struggle with correctly completing the long questionnaires. A standardized language test battery will be additionally administered at the post-intervention and follow-up assessments. Correlating its results with those of the parental questionnaires will increase the reliability of the obtained effects.

Furthermore, in an optimal scenario, a standardized behavioral battery of musical abilities would be included at all three assessment points to show reliable intervention-specific effects on the behavioral level in addition to the neural level. Similarly, in the best case, a general cognitive/developmental status evaluation would be conducted at all assessment points to evaluate the possible discrepancies between it and language development. However, due to time constraints caused by the young age of the participants and in order to not burden the families with an excessive amount of laboratory visits, the behavioral assessment was restricted to language (and motor) development for which standardized behavioral tests exist for this age group. To complement this information, any concerns or diagnoses related to the child’s development are inquired from parents at all assessment points.

The inclusion of multiple outcome variables in the present trial can be considered both a strength and a limitation. The inclusion of several primary variables increases the risk of underpowered analyses and overly positive conclusions on intervention efficacy. It is also important to note the limitations of the sample size in reliably probing all the secondary and exploratory outcomes. Nevertheless, the strength of the approach is that the behavioral and neural measures can provide complementary information on early language development and a broader view of the specific benefits of the intervention. The clearly larger sample size compared to previous similar studies (Benasich et al., 2014; Gerry et al., 2012; Musacchia et al., 2017; Zhao and Kuhl, 2016; Zhao et al., 2022) enables preliminary exploration of many aspects that have been overlooked in earlier studies, including, for example, the role of participant age on intervention efficacy. These findings can provide important starting points for future, more focused studies.

## 4 Conclusion

Active, social, and group-based musical activities are a motivating, feasible, and cost-effective means of supporting development at an early age. The present trial has the opportunity to provide novel evidence on their efficacy for early language development compared to other active and social activities. Importantly, it will shed light on essential questions related to who can benefit from music activities and what mechanisms underlie the positive effects. This will be achieved by investigating the participant-related factors contributing to intervention efficacy, including but not restricted to age, developmental stage, and genetic risk factors of the infants, as well as examining the role of social–emotional factors as mediators of intervention effectiveness. The results of this trial will thus help in planning effective and well-targeted group interventions for supporting early language development that could be recommended in both educational and clinical settings for infants and small children.

## Footnotes

1 This research question serves the purpose of confirming that both intervention arms are successful in filling their specific aims.

2 Direct investigation of change in the ERP and MMR response properties may not be always possible, because the morphology of the ERPs and MMRs (for example, the amount and polarity of obtained response peaks) is expected to differ markedly according to age group [see, e.g., (Thiede et al., 2019)]. However, whenever similar response peaks are observed in the two assessment points (i.e., expected to reflect the same ERP or MMR component), the change in their mean amplitude and peak latency will be analyzed between the two assessment points.

3 Choosing and quantifying the variables reflecting developmental status will be based on language and motor abilities at baseline, and may be treated as categorical (e.g., reached or not reached basic developmental milestones) or continuous (scores in the relevant questionnaires).
