# Supplementary material for: Language development deficits and early interactive music intervention (BusyBaby): protocol description of a double-blind randomized controlled trial on the effectiveness of music on language development in infancy
Source: Front Psychol. 2026 Jan 5;16:1699558. doi: 10.3389/fpsyg.2025.1699558 (PMC12812983; doi:10.3389/fpsyg.2025.1699558)
Supplement: Supplementary file 1 [file Data_Sheet_1.pdf]

Supplemental material

Music and circus interventions in the BusyBaby trial

1 Planning and principles of the interventions

The music and circus interventions were planned and conducted by two music teachers and two circus teachers, respectively, in collaboration with author PV. First, general principles and overall structure of the whole intervention including duration and number of intervention periods and sessions, were agreed on together (see section 2). Then the specific contents of the two interventions including learning goals, methods, specific songs/exercises, and equipment for all intervention sessions, were separately planned by the respective teachers and reviewed by PV (see section 3). The plans were made during spring and summer 2024 and thus finalized before the start of the first intervention groups in September 2024. Thus, the teachers adhered to the session-specific plans that they had designed together beforehand, and reported all possible deviations from the plans after each session (4.1).

In both interventions, the main learning methods revolved around creativity and play, and two important joint learning goals were 1) finding joy and pleasure in the art of music/circus, and 2) supporting positive interaction, both between the parent and the infant and within the whole intervention group. The latter included general goals such as learning to be part of a group, gaining a sense of belonging to the group, and basic social skills such as turn-taking and sharing.

In order to make a clear division between the methods adopted in the two intervention arms, as general principles it was agreed that 1) *joint singing and musical instruments* are only used in the music intervention, 2) dancing, moving, clapping, or drumming *to the beat of music* is only done in the music intervention, and 3) *physical exercise* including strength and balance training and acrobatics is only done in the circus intervention. Both intervention arms were allowed to include recorded music, single spoken nursery rhymes, physical closeness including holding, touching, rocking, and bouncing the infant, as well as relaxation periods. However, these activities served partly different aims in the two intervention arms as described in section 3.

2 Intervention and session structure

The intervention consists of 20 sessions that are thematically organized to five consecutive periods including four sessions each; the four sessions within a period have approximately the same overall

contents (including the general theme, specific songs/exercises, equipment, and learning goals). The intervention instructors have a choice to always introduce the periods in the same order, or vary the order between intervention groups. The themes of the five four-week periods are forest, colors, vehicles, birds, and water (in the music intervention)/space (in the circus intervention).

Irrespective of the period and intervention arm, the intervention sessions have the same general structure consisting of the following five activities in a fixed order: 1) a beginning ritual (5–10 min), 2) warm-up/movement (10–15 min), 3) main learning goal (10–15 min), 4) unstructured time (10–15 min; including familiarizing with equipment, socializing within the group, and relaxation), and 5) an ending ritual (5–10 min). While the beginning and ending rituals are always the same within the intervention (but not between music and circus interventions), the content and equipment in the other activities vary between the five training periods and, to a smaller extent, evolve during each period as the skill level and confidence of the participants increases with repetition. The intervention and session structure are illustrated in Figure 1.

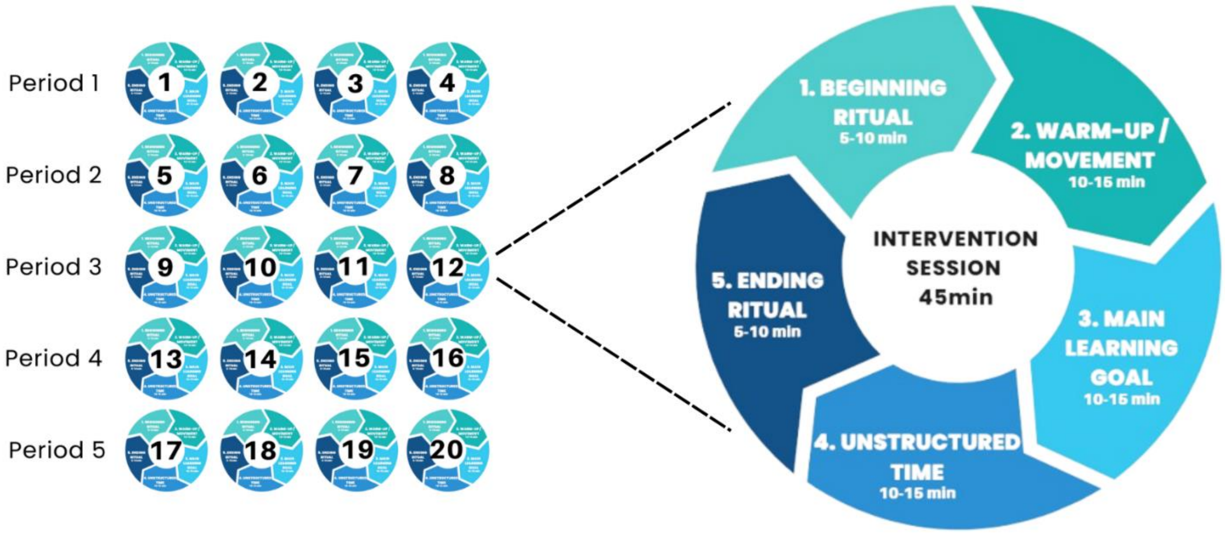

Figure 1. Intervention and session structure in the BusyBaby trial. The intervention consists of 20 sessions organized to five periods with four sessions each (left). Each intervention session has the same general structure (right).

3 Intervention contents

3.1 Music intervention: learning goals, content, equipment, and venues

The arm-specific learning goals in the music intervention are focused on basic musical skills. These include exploring sounds, observing and developing a steady, simultaneous musical beat together as a group, to learn and gain awareness of musical forms, rhythms, and melodies, and to familiarize with diverse musical genres and Finnish children's song repertoire. Main elements in all sessions include singing, playing musical instruments, moving to music, and listening to music. Parents are encouraged to participate actively. New songs are learned from recorded music or from teacher speaking or singing them verse by verse and asking the parents to repeat. Joint singing (mostly parents) is emphasized, and it is done both with accompaniment (teacher accompanies singing with piano, ukulele, or kantele) and without accompaniment, often together with playing percussion instruments (parents and infants together).

All music intervention sessions follow approximately the following structure. At the start of each session, as a beginning ritual, the teacher, parents, and infants gather to a circle and the infants are welcomed by a song where all their names are gone through one by one. The teacher sings and plays an instrument for accompaniment, and the parents are encouraged to sing along. During the following warm-up section, the infants and parents can freely move in the room while they sing a warm-up song coupled with different movements (clapping, walking, jumping etc.). The group then sits down again for the main learning goal. After a play song, a new song is introduced and its melody and lyrics are learned together with repetition. The song is repeated and paired with one or two different musical instruments. An unstructured relaxation section follows, where recorded music is coupled with, for example, soap bubbles or colorful scarves. The ending ritual is a goodbye song coupled with a choreography of hand movements.

Live music is mainly used in the music intervention instead of recorded music. Recorded music is mainly utilized for relaxation (background music to set the mood) or for learning a new song before singing and playing it together. Spoken nursery rhymes are used regularly for learning song lyrics and the beat and rhythm of the song. They are typically combined with rhythmic, playful bodily exercises such as tapping on the infant's body parts or bouncing the infant, or with playing a musical instrument. Motor exercises in the music intervention are always done rhythmically to the beat of

music or nursery rhymes and mainly serve the purpose of learning to perceive and produce sound and rhythm, either with musical instruments or with body percussions such as clapping or marching.

Equipment includes the following musical instruments: wooden xylophone bars and mallets, maracas, claves, djembe drums, frame drums, and a selection of other small percussion instruments such as triangles and egg shakers. The instruments are usually introduced one at a time during the sessions and collected away after use. In most cases both the infant and the parent are given their own instrument. Additional equipment is used according to the theme of the intervention period and activity; this can include stuffed animals, toy cars, feathers, scarves, and a rainbow parachute. The teacher additionally plays piano, ukulele, or kantele during the sessions as accompaniment. The music interventions are held in two regional music schools located in Helsinki, Finland, in classrooms designed for family and children's music playschool classes. The size of the classrooms is around 30–40 m^2 (320–430 square feet).

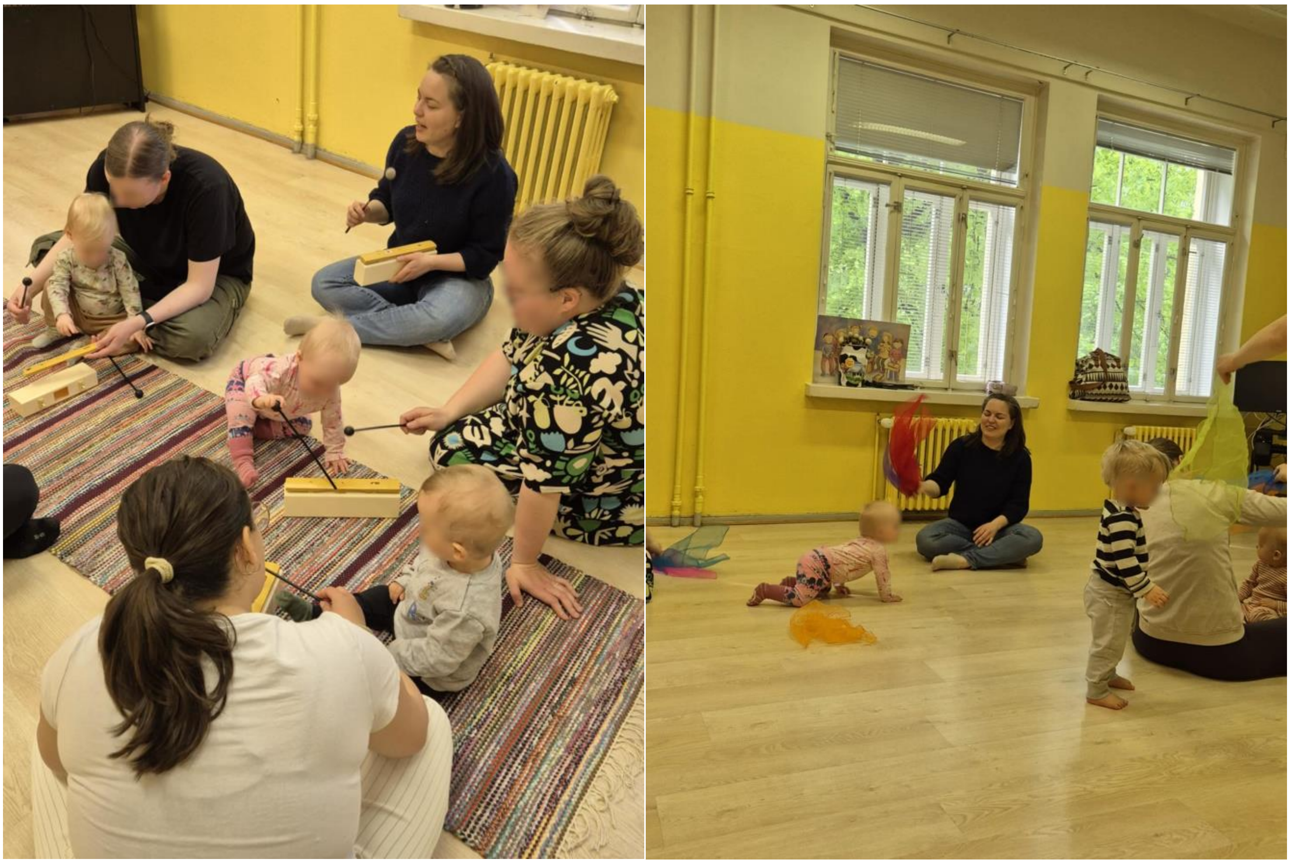

Figure 2. Learning to play a xylophone bar (left) and unstructured time (right) in a music intervention. The intervention venue is Central Helsinki Music Institute in Helsinki, Finland. Parents have provided written consent for publication of photos taken in the intervention groups where they or their child are visible (so that no faces are shown; consent form provided by the University of Helsinki).

3.2 Circus intervention: learning goals, content, equipment, and venues

The arm-specific learning goals in the circus intervention are focused on motor skills and bodily control and awareness of both the infant and the parent, as well as on supporting their bodily interaction. Motor skills include those related to whole-body movement (e.g., running, jumping), handling skills (e.g., holding, throwing and catching), and balance. Main elements in all sessions include acrobatics and other age-appropriate motor exercises for the parent and infant, and familiarizing with the art and equipment of circus and acrobatics. The acrobatics exercises revolve around the parent holding and balancing the infant in different ways and increase in difficulty as the dyads progress during the intervention. Other motor exercises consist of moving in different ways such as walking, running, crawling, and jumping, balancing exercises, as well as exercises where the parent rocks, throws and catches, or spins the infant. The parents may practice circus techniques such as juggling, balancing, and aerial acrobatics together with their infants, or on their own while the infants freely explore the equipment on the floor. The equipment may also be organized by the teacher to trick tracks or obstacle courses that the infants can complete with the help of their parents.

All circus intervention sessions follow approximately the following basic structure. As a beginning ritual, the group sits on the floor around the rainbow parachute and the teacher goes through the names of all infants and parents present and welcomes them by asking something related to the session theme. This is followed by the welcome rhyme, recited together by the teacher and parents and combined with playful and soothing massage and movement on different body parts of the infants by their parents. The teacher then asks the parents to stand up for a warm-up exercise. The infants may participate from the floor level or be held by their parents. The warm-up is followed by a pair acrobatics exercise, which is the main learning goal for the session. After a spoken nursery rhyme coupled with playful movement and massage of the infants by their parents, the teacher first shows the exercise with a mascot (stuffed animal), and then goes around the room instructing all parent-infant dyads while they try it out. During the following unstructured time, the parents and infants are given the opportunity to freely examine and try out circus equipment. After a short clean-up together, each session ends with an ending ritual similar to the beginning rhyme, with the teacher, parents, and infants sitting on the floor around the rainbow parachute.

Only recorded, not live music, is used in the circus interventions, with the main purpose of setting the mood or introducing the theme of the session. The recorded music is soft, and not accompanied

by movements synchronized to music or joint singing, but it may be used in the background during the warm-up/movement sections. Spoken nursery rhymes are used in the beginning and ending rituals and as an introductory ritual to the pair acrobatics exercises, with the purpose of story-telling and making the bodily exercises into a play.

Equipment in the circus intervention sessions includes the following: ball pit balls, juggling balls, juggling rings, juggling scarves, diabolos, hoops, balance balls and tight wires, balance boards (rola bola), aerial hammocks, trapezes, aerial hoops, and aerial net hammocks. A wide selection of equipment is available during all sessions typically during the unstructured time, and the infants and parents can familiarize with them freely. The amount and variety of the equipment increases as the intervention progresses. The teacher shows an example for how to try out the equipment, and guides the parents and infants. The circus interventions are held in two private circus schools located in Helsinki, Finland, in classrooms designed for family and children's circus lessons. The floor of the classrooms is a sprung or rubber floor, and additional mattresses are available for the exercises as needed. The size of the classrooms is around 80–90 m^2 (860–970 square feet).

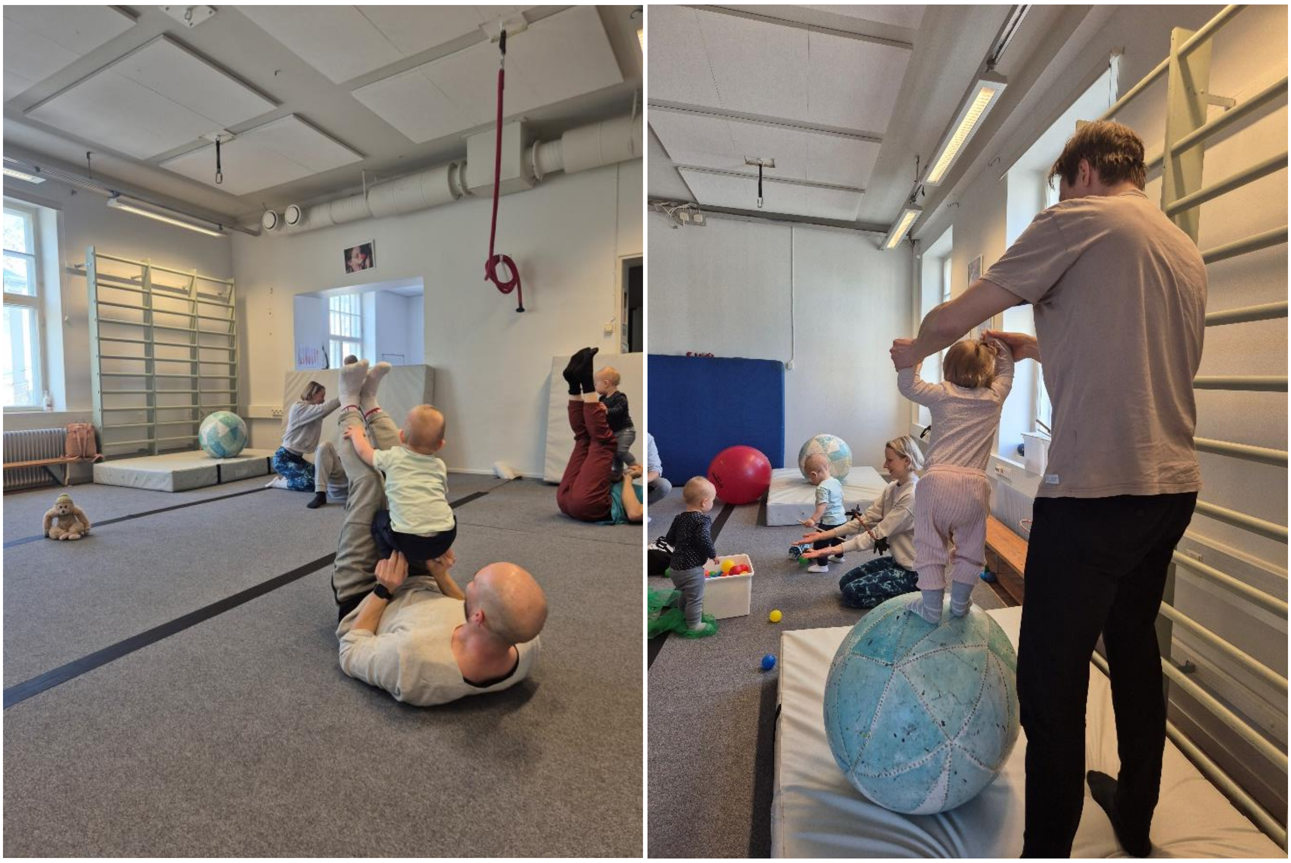

Figure 3. Pair acrobatics exercises (left) and unstructured time (right) in the circus intervention. The intervention venue is Circus Helsinki, Helsinki, Finland. Parents have provided written consent for publication of photos taken in the

intervention groups where they or their child are visible (so that no faces are shown; consent form provided by the University of Helsinki).

3.3 Social structure of the two intervention arms

It is relevant to note that while both intervention arms consist of structured lessons with specific learning goals, the social structure of the two intervention arms has differences that stem from the method itself (music vs. circus). While music making requires joint, simultaneous, and paced activity, circus exercises do not require this temporal synchrony and are therefore performed in a more individual pace within each parent-infant dyad. This individual pace also allows for more infant-initiated activities (what is done, when, and how many times) and, on the other hand, more parent-initiated activities, i.e., activities that the parent can choose freely when the infant is occupied with their own activity. This typically happens less in the music intervention lessons. Offering the parents and infants the opportunity to freely familiarize with the circus equipment without formal instructions is a typical part of a family circus lesson, while this is less common in a musical playschool setting.

To this end, to acknowledge this different social structure of the intervention arms and its potential implications for intervention outcomes, it was agreed that both intervention arms would include all of the following types of social activities in all lessons, however with intervention-specific variability in their relative amounts: 1) joint activity, where the whole group is engaged in the same activity either simultaneously or by taking turns, 2) dyadic activity, where each parent-infant dyad is engaged in their own activity according to teacher's instructions, and 3) socially unstructured activity, where parents and infants have opportunities to interact with each other without a joint task. Importantly, the relative amounts of these three types of social activity are tracked after each lesson with a questionnaire, so that they can be taken into account in data analyses (see section 4.1).

3.4 Intervention instructors

All intervention groups are run by one experienced instructor, and both intervention arms have two teachers who teach 4–6 groups each (estimate; out of a total of 10 groups per arm). The instructors within the same intervention (music/circus) occasionally act as substitutes for each other, in order to avoid cancelling sessions; no other substitute teachers will be hired.

Instructors in the music intervention are two teachers with a degree in early childhood music education (BA level) and five and six years of experience in teaching early childhood music education (children 0–6 years). Instructors in the circus intervention are two teachers, each with four years of experience in teaching circus to children 0–6 years of age. While a higher-education degree in circus teaching is rare and a specific degree in family circus is not available in Finland, both of the circus instructors have completed shorter courses and workshops on family circus. One of the instructors has a degree in dance pedagogy (BA level), while the other has over 10 years of teaching experience in a circus school.

4 Intervention-related questionnaires

4.1 Instructor questionnaire: session diary

After each session, intervention instructors fill in a session diary in REDCap (Research Electronic Data Capture), including a list of participants and a session report. The list of participants includes information on the infants' attendance/nonattendance and who accompanied them in the session. In case the infant was absent for the whole session and depending on whether their parent informed the instructor on the reason for nonattendance, the reasons are reported to the session diary as illness, trip, other commitment, other (specify), or unknown. In case the infant was absent for a part of the session, the duration of attendance and the reason for partial nonattendance are reported. Intervention adherence is quantified from these sessions diaries and taken into account in the data analyses as a potential moderator of the intervention outcomes. The instructors additionally report in case their blindness regarding an infant's risk status (dyslexia risk or no risk) was violated during the session.

In the session report, the instructors report the actual duration of the session, whether they adhered to the session plan (and if not, how it was violated), whether other people than the participants and instructor were present (who), the duration of different social activities during the session (joint activity, dyadic activity, and socially unstructured activity, see 3.3), unexpected events and adverse effects (on free fields), and atmosphere during the session (rated on a sliding Likert-scale from "Exceptionally good" to "Exceptionally bad"). Relative amounts of the types of social activity as well as average atmosphere may be taken into account in data analysis as potential moderators of intervention outcomes. Reported adverse effects can include, for example, accidents, strong negative affect, or conflicts between group members including those within parent-infant dyads.

4.2 Parental questionnaire

After the last intervention session, all parents who participated in the intervention sessions more than once are asked to fill in a 13-item questionnaire in RedCap on their views regarding the intervention (Table 1; i.e., filled by one parent or both parents separately, depending on the participants). In the items, the parents rate their own attendance rate and the family's adherence to the intervention, as well as their motivation towards the intervention, including feelings towards the intervention, expected benefits for their infant and themselves, as well as their preference for this vs. the other intervention arm (all items marked with an asterisk in Table 1). This motivation towards the intervention is expected to moderate intervention outcomes and will be taken into account in the data analysis. We plan to calculate a combination score of these items. When both parents fill in the questionnaire, the score will be a weighted average of the two ratings, based on the relative amounts of attended sessions (based on the parental attendance rate confirmed from the instructor's session diary, see 4.1).

Table 1. Content of the parental questionnaire. All items except item 11 were rated on a 5-point Likert scale (numerically from 2 to -2 or vice versa). Items marked with an asterisk (*) are assumed to reflect motivation towards the attended intervention.

Item	Variable name	Item	Response scale
1	Attendance rate, parent	I attended the sessions with the child	5-item Likert (always or almost always/only occasionally)
2*	Liking, parent	I liked attending the sessions	5-item Likert (strongly disagree/agree)
3*	Liking, child	My child seemed to like attending the sessions	5-item Likert (strongly disagree/agree)
4*	Benefit, child development	Attending was beneficial for my child's development	5-item Likert (strongly disagree/agree)
5*	Benefit, parent well-being	Attending was beneficial for my well-being	5-item Likert (strongly disagree/agree)
6*	Benefit, child well-being	Attending was beneficial for my child's well-being	5-item Likert (strongly disagree/agree)
7*	Benefit, interaction	Attending had a positive effect on the relationship between me and my child	5-item Likert (strongly disagree/agree)

8*	Benefit, language development	Attending was beneficial for my child's language development	5-item Likert (strongly disagree/agree)
9*	Benefit, motor development	Attending was beneficial for my child's motor development	5-item Likert (strongly disagree/agree)
10	Adherence	How regularly did you attend the sessions?	5-item Likert (seldom or never/always or almost always attended)
11	Low adherence, reasons	If you did not always attend the sessions, what were the main reasons for not attending?	Choose one or several (illnesses, lack of motivation, other commitments, exhaustion/lack of resources, unsuitable time or location, other reasons – specify)
12*	Arm preference	The trial had two different intervention arms. Were you happy with your assignment to this arm?	5-item Likert (clearly preferred other arm/this arm)
13*	Arm benefits	Thinking of your assignment to this arm, how do you feel about its benefits to your child, yourself, and the relationship between you and your child?	5-item Likert (clearly more benefits from other arm/this arm)
